# SPP1 (osteopontin): a key regulator orchestrating cancer progression and non-cancerous pathologies - therapeutic opportunities and future directions

**DOI:** 10.3389/fcell.2026.1912468

**Published:** 2026-08-05

**Authors:** Bo Pang, Huan Sun, Mingyu Ouyang, Cong Hu

**Affiliations:** 1 Cardiology Department, First Hospital of Jilin University, Changchun, Jilin, China; 2 Electrodiagnosis Department, Qianwei Hospital of Jilin Province, Changchun, China; 3 Department of Reproductive Medicine, Hezhou People’s Hospital of Guangxi Zhuang Autonomous Region, Hezhou, China; 4 Reproductive Medicine Center, Prenatal Diagnosis Center, First Hospital of Jilin University, Changchun, Jilin, China

**Keywords:** angiogenesis, epithelial-mesenchymal transition, pregnancy, secreted phosphoprotein 1, tumor

## Abstract

Secreted Phosphoprotein 1 (SPP1) is a multifaceted matricellular glycoprotein that mediates diverse biological processes via integrin and CD44 receptor signaling. While its profound contributions to tumor progression and metastasis are extensively documented, emerging evidence underscores its pivotal roles across diverse non-cancerous conditions, including pregnancy-related complications, fibrotic diseases and neurological disorders. Crucially, SPP1 exhibits functional pleiotropy, orchestrating angiogenesis and epithelial-mesenchymal transition, while modulating immunosuppression. These collective actions drive disease progression, establishing SPP1 as a compelling therapeutic target. This review synthesizes current understanding of SPP1 biology, highlighting mechanistic insights imperative for translating its therapeutic potential into safe, precision-targeted interventions against diverse SPP1-driven pathologies.

## 1 Introduction

Secreted phosphoprotein 1 (SPP1), also known as osteopontin (OPN), is a multifunctional phosphorylated glycoprotein that has emerged as a pivotal molecule in a wide array of physiological and pathological processes (Yan et al., 2023). Initially identified as a bone matrix protein crucial for osteoclast attachment and bone remodeling, SPP1 is now widely recognized as a versatile cytokine and extracellular matrix component. It is expressed by numerous cell types, including osteoblasts, macrophages, T cells, endothelial cells, and various cancer cells (Ellert-Miklaszewska et al., 2025; Li et al., 2025; Gao et al., 2022). Structurally, SPP1 contains an arginine-glycine-aspartate motif that mediates binding to several integrins, as well as a CD44-interacting domain, enabling it to engage a wide range of cell surface receptors and activate context-dependent downstream signaling pathways (Hou et al., 2025). The structural versatility underpins the capacity of SPP1 to regulate cell adhesion, migration, survival, and differentiation across different biological contexts (Jiang et al., 2026; Liu L. et al., 2022; Lei et al., 2025).

Recent advances in single-cell RNA sequencing and spatial transcriptomics have greatly refined our understanding of cellular heterogeneity in both tumors and non-malignant diseased tissues, highlighting SPP1 high expressing subpopulations as functionally distinct mediators of disease progression (Wang et al., 2023; Rakina et al., 2024; Xiao et al., 2025). SPP1 contributes to malignant progression through multiple mechanisms, including promoting cell proliferation, inhibiting apoptosis, enhancing invasion and metastasis, and modulating the tumor microenvironment (Tan et al., 2022; Panda et al., 2024). Notably, SPP1^+^ macrophages and myeloid-derived suppressor cells (MDSCs) have emerged as recurrent and clinically relevant immune subsets across multiple tumor types, which are associated with immunosuppression, matrix remodeling, metastatic dissemination, and poor prognosis (Wang et al., 2025; Liang et al., 2026; Lu et al., 2021). These findings position SPP1 not merely as a downstream biomarker, but also an active regulator of tumor cell plasticity and tumor-stroma crosstalk. Beyond oncology, SPP1 also exerts essential functions in a variety of non-cancerous conditions, particularly involving tissue remodeling, fibrosis, inflammation, and reproductive biology. In normal reproductive physiology, SPP1 is critically involved in embryo implantation, maternal-fetal communication, and placental development (Pan et al., 2019; Shevde and Samant, 2014; Subraman et al., 2015). In parallel, aberrant SPP1 signaling has been increasingly linked to fibrotic diseases and chronic inflammatory disorders, suggesting that SPP1 may serve as a common molecular link developmental regulation, immune activation, and pathological tissue repair (Ji et al., 2023). Given its broad biological functions and disease relevance, SPP1 has attracted increasing attention as both a biomarker and a therapeutic target. Preclinical studies have explored multiple strategies to inhibit SPP1 signaling, including neutralizing antibodies, RNA interference, aptamers, and receptor-targeting approaches, with encouraging results in models of cancer and fibrosis (Weber, 2026; Li et al., 2026; An et al., 2023). Future research will be crucial in dissecting the nuanced molecular mechanisms by which SPP1 orchestrates disease progression, developing more refined therapeutic strategies, and exploring its potential as a diagnostic biomarker and a predictive tool for treatment response across diverse conditions (Box 1).

Box 1 **Clinical Significance of SPP1 Research**

Diagnostic and Prognostic Biomarker: Elevated SPP1 levels in tumor tissue, serum, plasma, urine, and cerebrospinal fluid are associated with advanced disease stage, higher tumor grade, metastasis, and reduced overall survival across multiple cancer types. SPP1 also holds promise as a non-invasive biomarker for pregnancy complications fibrotic disease and neurological disorders progression.

Immune Microenvironment Modulator: SPP1^+^ macrophages constitute a clinically relevant immunosuppressive subset that promotes T-cell exhaustion, recruits regulatory immune cells, and facilitates immune evasion. The abundance of SPP1^+^ macrophages correlates with poor responses to immune checkpoint inhibitors, suggesting SPP1 as a predictive biomarker for immunotherapy.

Therapeutic Target: Preclinical studies have demonstrated that disrupting the SPP1/integrin/CD44 axis via neutralizing antibodies, aptamers, RNA interference, or small-molecule inhibitors can suppress tumor angiogenesis, reverse epithelial-mesenchymal transition, attenuate metastasis, and reinvigorate antitumor immunity. SPP1 inhibition also reduces pathological fibrosis in preclinical models of pulmonary, hepatic, and endometrial fibrosis.

Distinct from prior reviews focused on oncology, this review addresses a key knowledge gap by interrogating how conserved SPP1 signaling orchestrates both physiological adaptation and pathological progression. Building on this mechanistic insight, we further discuss the value of SPP1 as a diagnostic and prognostic biomarker and evaluate current therapeutic strategies. Ultimately, this comprehensive overview aims to establish a robust framework that will accelerate the translation of SPP1 biology into precision medicine.

## 2 Methods

A comprehensive literature search was conducted across three major databases: PubMed, Web of Science, and Scopus. The search strategy employed a combination of Medical Subject Headings and free-text keywords, including “SPP1,” “osteopontin,” “OPN,” “neoplasm,” “cancer,” “angiogenesis,” “epithelial-mesenchymal transition,” “metastasis,” “immunosuppression,” “pregnancy,” “fibrosis,” “neurological disorders,” “neuroinflammation,” “multiple sclerosis,” “Alzheimer’s disease,” “Parkinson’s disease,” “stroke,” and “therapeutics.” The search was restricted to articles published between January 2000 and May 2026. Inclusion criteria comprised original research articles and relevant reviews focusing on SPP1 biology and its clinical implications in human health and disease. Conference abstracts, case reports, and non-English publications were excluded. The selection process involved an initial title and abstract screening, followed by a full-text review of potentially eligible studies.

## 3 The role of SPP1 in tumor progression

SPP1 expression has been documented in a wide range of malignancies, including colorectal cancer, hepatocellular carcinoma, breast cancer, lung cancer and glioblastoma (Qi et al., 2022; Liu Y. et al., 2022; Gu et al., 2024; Mei et al., 2025; Jia et al., 2016). A substantial mount of evidence has established SPP1 as a key driver of tumor progression through multiple mechanisms, orchestrating a complex interplay of cellular and molecular events that profoundly impact several hallmarks of cancer. Specifically, SPP1 has been implicated in promoting sustained proliferative signaling, enhancing resistance to cell death, facilitating epithelial-mesenchymal transition (EMT), stimulating angiogenesis, and critically, remodeling the tumor microenvironment (TME) to foster an immunosuppressive and pro-metastatic milieu. The functional significance of SPP1 extends to virtually every critical aspect of cancer biology, from initial primary tumor growth and local invasion to distant metastatic dissemination and therapeutic resistance, making it a compelling target for in-depth investigation.

### 3.1 Angiogenesis

Angiogenesis, the formation of new blood vessels from pre-existing vasculature, is a fundamental process in both physiological development and pathological conditions, particularly cancer (Larionova et al., 2021). The clinical relevance of SPP1-driven angiogenesis is supported by studies showing that elevated SPP1 levels in tumor tissues or plasma are associated with increased microvessel density and poor prognosis across multiple cancer types, acting both directly on endothelial cells and indirectly by regulating other angiogenic mediators (Long et al., 2024; Yu et al., 2023).

Mechanistically, SPP1 exerts direct pro-angiogenic effects on endothelial cells. In human umbilical vein endothelial cells, SPP1 activates the PI3K/AKT signaling pathway, leading to upregulation of anti-apoptotic proteins such as Bcl-xL and activation of nuclear factor-κB (NF-κB), thereby enhancing endothelial cell survival during angiogenesis (Dai et al., 2009). In addition, the interaction between SPP1 and integrins on endothelial cells is essential for mediating these angiogenic effects, especially α_v_β_3_ integrin (Rodrigues et al., 2007). In glioblastoma, SPP1^+^ macrophages have been shown to promote pathological angiogenesis, with Recombinant A Disintegrin And Metalloprotease 8 (ADAM8) acting as a key regulator via the JAK/STAT3 pathway (Li et al., 2021). In hepatocellular carcinoma (HCC), SPP1 expression is not only significantly associated with vasculogenic mimicry but also activates the Colony-Stimulating Factor1 (CSF1)-CSF1R pathway, thereby facilitating macrophage recruitment and forming a positive feedback loop that further enhances angiogenic signaling (Li et al., 2025).

**FIGURE 1:**
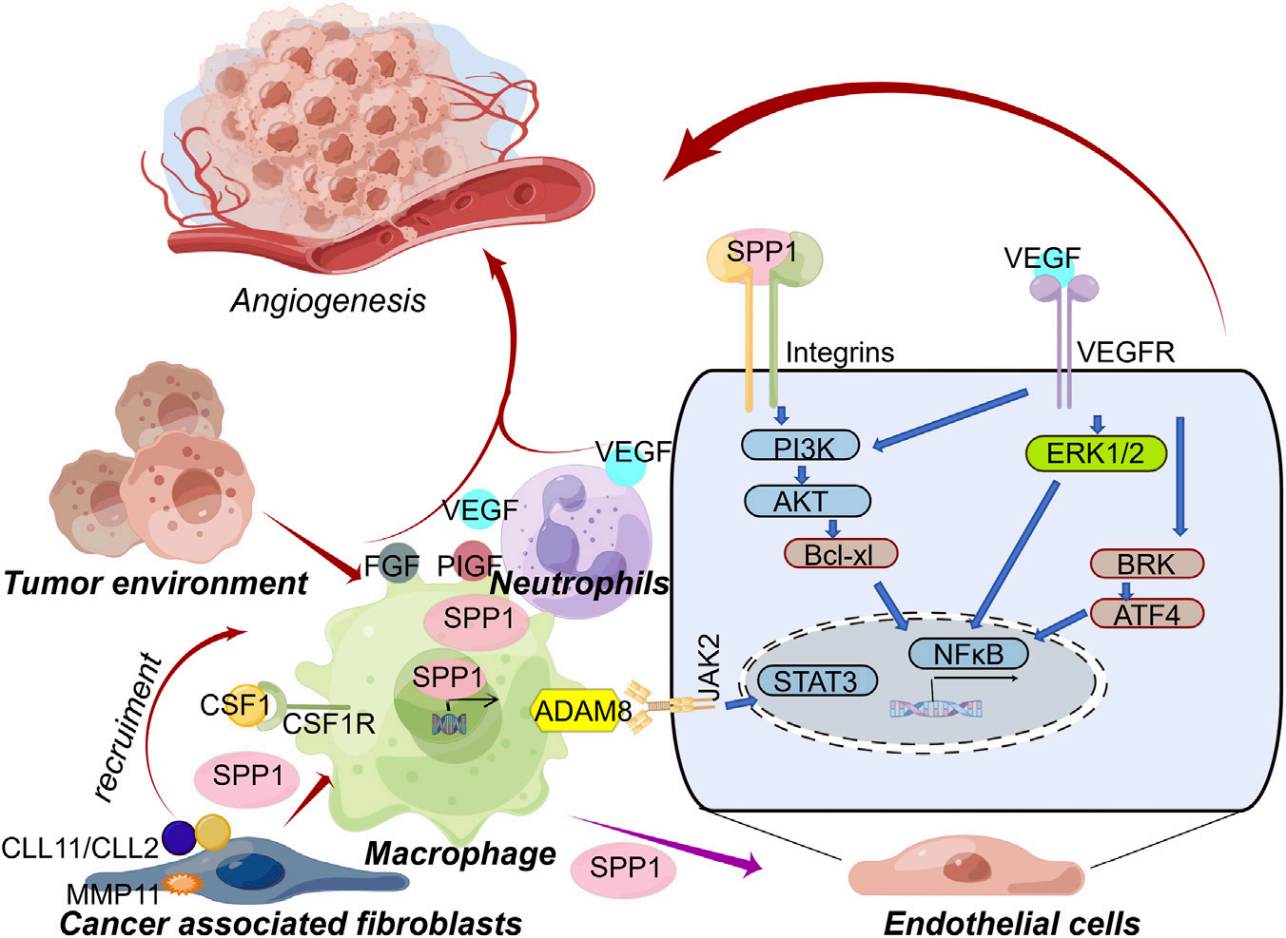
SPP1-Mediated Regulation of Tumor Angiogenesis. SPP1 activates PI3K/AKT, NF-κB, and integrin-mediated signaling in endothelial cells, thereby enhancing endothelial survival and angiogenic activity. SPP1^+^ macrophages further contribute to pathological angiogenesis through ADAM8-mediated activation of the JAK/STAT3 pathway. SPP1 also activates the CSF1/CSF1R axis, promoting macrophage recruitment and establishing a positive feedback loop that amplifies angiogenic signaling. SPP1 also induces VEGF expression via the BRK/NF-κB/ATF4 signaling axis or colocalize with VEGFA^+^ neutrophils to strengthen angiogenesis. MMP11^+^ cancer associated fibroblasts recruit SPP1^+^ macrophages through CCL11/CCL2 signaling, thereby enhancing VEGFA secretion. SPP1, secreted phosphoprotein 1; PI3K, phosphoinositide 3-kinase; AKT, protein kinase B; NF-κB, nuclear factor kappa-light-chain-enhancer of activated B cells; ADAM8, A disintegrin and metalloprotease 8; JAK, janus kinase; STAT3, signal transducer and activator of transcription 3; CSF, colony-stimulating factor; VEGF, vascular endothelial growth factor; BRK, breast tumor kinase; ATF4, activating transcription factor 4; MMP, matrix metalloproteinase; CCL2, C-C Motif Chemokine Ligand 2.

Another major mechanism by which SPP1 promotes angiogenesis is through the induction of vascular endothelial growth factor (VEGF), one of the most potent and specific angiogenic factors. In breast cancer, SPP1 promotes VEGF-dependent tumor growth and angiogenesis through activation of the breast tumor kinase/NF-κB/activating transcription factor-4 signaling axis. The induced VEGF subsequently acts in an autocrine manner on endothelial cells to further activate PI3K/AKT and ERK1/2 signaling, thereby strengthening the angiogenic response (Dai et al., 2009). Beyond its direct effects, SPP1 also shapes the angiogenic microenvironment through SPP1^+^ macrophages. SPP1^+^ macrophages secrete a broad repertoire of pro-angiogenic mediators, including VEGF, fibroblast growth factor, and placental growth factor, which collectively promote neovascularization (Larionova et al., 2021; Bu et al., 2021). Notably, a recent single-cell transcriptomic study unveal that in triple-negative breast cancer, SPP1^+^ macrophages colocalize with VEGFA^+^ neutrophils and endothelial tip cells in hypoxic regions, forming a specialized pro-angiogenic microenvironment (Choi et al., 2025). Similarly, in bladder cancer, matrix metalloproteinase (MMP)11^+^ cancer-associated fibroblasts (CAFs) recruit SPP1^+^ macrophages through CCL11/CCL2 signaling, after which the recruited macrophages promote VEGFA secretion and enhance the pro-angiogenic activity of tip endothelial cells (Xu W. et al., 2025). Co-expression of SPP1 and VEGF has been observed in multiple pathological settings, and accumulating evidence suggests reciprocal regulation between the two molecules (Ramchandani and Weber, 2015). However, anti-VEGF antibodies only partially suppress SPP1-induced angiogenesis by interrupting this downstream loop, whereas anti-SPP1 antibodies can completely abolish the biological effects, suggesting that SPP1 functions as a more upstream and broader regulator of angiogenesis (Dai et al., 2009). In certain tumors, SPP1 may also promote angiogenesis through VEGF-independent pathways, rendering these tumors less responsive to VEGF-targeted therapies (Larionova et al., 2021) (Figure 1).

### 3.2 Epithelial-mesenchymal transition (EMT), and invasion

EMT is a fundamental cellular reprogramming process in which epithelial cells lose their polarity and cell-cell adhesion characteristics and acquire a mesenchymal, migratory phenotype (Kothari et al., 2016). While EMT is essential for embryonic development and wound healing, its aberrant activation is a hallmark of cancer progression, facilitating invasion, metastasis, and therapeutic resistance (Demirkan, 2013).

SPP1 promotes EMT through the upregulation of key EMT-associated transcription factors. Mechanistically, SPP1 activates Twist through serine phosphorylation, a post-translational modification critical for its transcriptional activity and subsequent induction of EMT-related genes, which repress E-cadherin expression and induce mesenchymal markers such as vimentin and N-cadherin (Ny et al., 2013). Similarly, in gastric cancer and HCC, SPP1 promotes EMT through the PI3K/AKT/mTOR signaling cascade, enhancing cell proliferation, invasion, and migration (Yu et al., 2018; Qin et al., 2023). The ERK1/2 pathway also plays a crucial role in SPP1-mediated EMT, as demonstrated in endometrial cancer cells where SPP1 activates both AKT and ERK1/2 signaling to promote EMT and invasive behavior (Li et al., 2015).

Beyond its direct effects on cancer cells, SPP1 significantly modulates its receptors, particularly integrins and CD44. SPP1 binding to integrin αvβ3 activates downstream signaling cascades, including PI3K/AKT and ERK, which converge on EMT transcription factors. In pancreatic cancer, hypoxia-driven paracrine SPP1 signaling from pancreatic stellate cells interacts with integrin αvβ3 on cancer cells to upregulate forkhead box protein M1, thereby inducing EMT and cancer stem cell-like properties (Cao et al., 2019). In colorectal cancer, SPP1^+^ macrophages interact with cancer cells through the SPP1-CD44, SPP1-PTGER4, and SPP1-α4β1 complex axes to amplify EMT and enhance migration and invasion (Xie et al., 2025; Ding et al., 2025). SPP1 also regulates EMT through interactions with other key signaling pathways. The TGF-β pathway, a canonical EMT inducer, exhibits significant crosstalk with SPP1 signaling (Weber et al., 2012). In hepatitis C virus-associated HCC, SPP1 activation leads to increased phosphorylation of AKT and GSK-3β, followed by β-catenin activation, which promotes EMT of hepatocytes (Iqbal et al., 2013). In colorectal cancer, SPP1^+^ macrophages have been shown to induce EMT in cancer cells through the activation of JAK2/STAT3 signaling (Yang S. et al., 2025). Besides that, SPP1 enhances the expression and activity of MMPs, particularly MMP-2 and MMP-9, which degrade ECM components and facilitate cancer cell invasion (Qin et al., 2023). In gastric cancer, SPP1 upregulates MMP-9 and MMP-2 through activation of the PI3K/AKT pathway and hypoxia-inducible factor-1α (HIF-1α) (Song et al., 2009). On the other hand, HIF-1α directly regulates SPP1 transcription in macrophages, and the acidic tumor microenvironment synergistically increases SPP1 expression through HIF-1α/STAT3 signaling. This hypoxia-driven SPP1 upregulation in tumor associated macrophages (TAMs) promotes M2-like polarization and enhances the ability of these cells to induce EMT in cancer cells (Qu et al., 2025). In HCC, SPP1 knockdown results in decreased expression of MMP-2 and urokinase-type plasminogen activator, impairing the ability of cancer cells to form vasculogenic mimicry structures and invade surrounding tissues (Liu et al., 2011). The regulation of MMPs by SPP1 is also mediated through its interaction with integrin receptors, particularly αvβ3, which activates focal adhesion kinase and downstream signaling pathways that control cell motility and invasion (Chen et al., 2022). The influence of SPP1 on EMT and invasion extends beyond its direct effects on cancer cells to include modulation of the tumor microenvironment. Tumor-derived SPP1 can differentiate resident fibroblasts into CAFs, which in turn secrete CXCL12 to promote EMT in cancer cells (Butti et al., 2021). This paracrine loop establishes a feed-forward mechanism that sustains the invasive phenotype.

**FIGURE 2:**
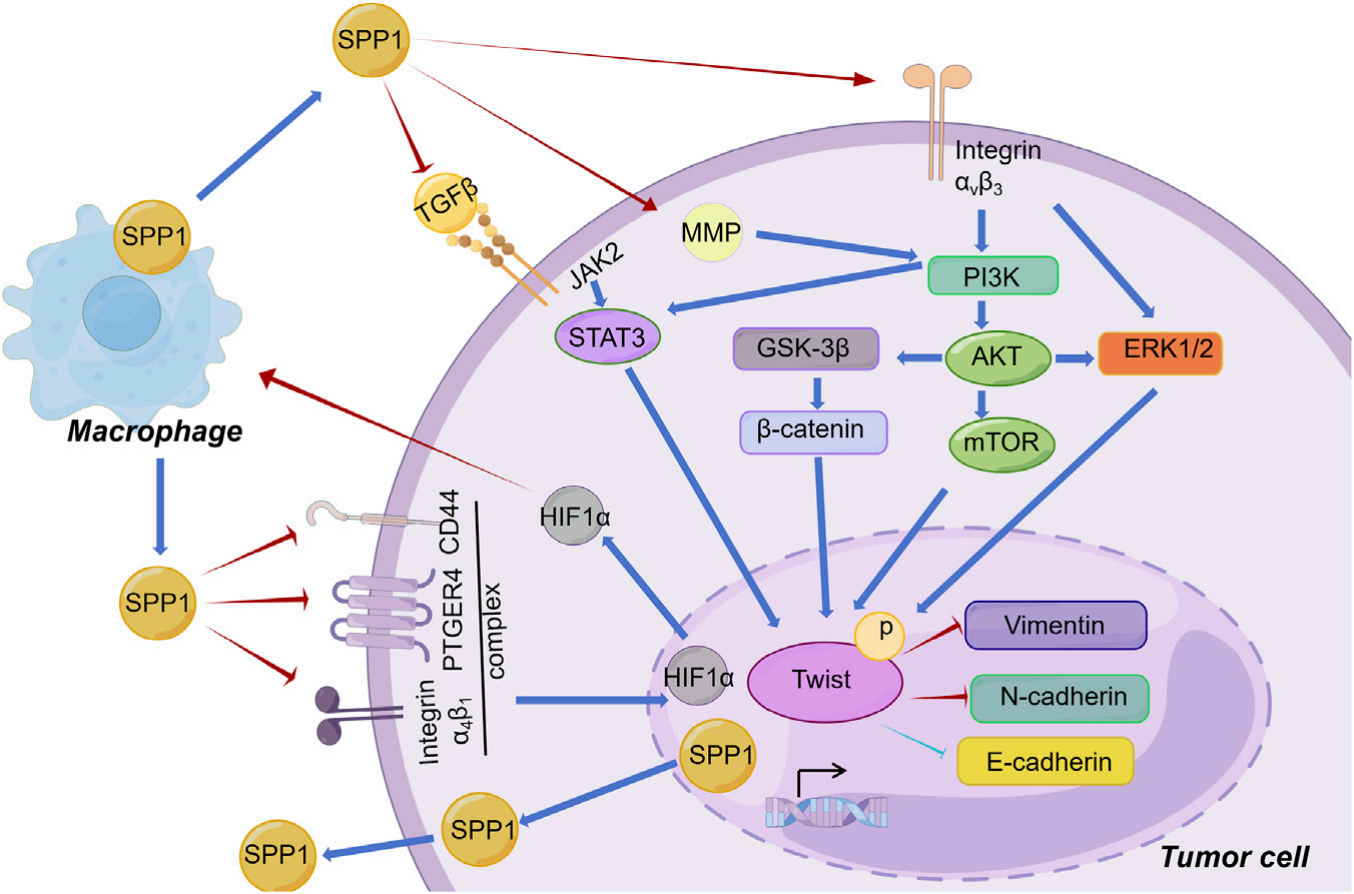
SPP1-Mediated Regulation of Tumor EMT. In cancer cells, SPP1 activates Twist, thereby inducing EMT-related transcriptional programs, repressing E-cadherin, and upregulating vimentin and N-cadherin. SPP1 further promotes EMT through activation of PI3K/AKT/mTOR, AKT/ERK1/2, and AKT/GSK-3β/β-catenin signaling pathways. SPP1-mediated engagement of integrins, particularly integrinα_v_β_3_, activates downstream PI3K/AKT and ERK signaling, converging on EMT transcription factors. In the tumor microenvironment, SPP1^+^ macrophages enhance EMT through SPP1–CD44, SPP1–PTGER4, and SPP1–α4β1 axes. SPP1 also crosstalks with TGF-β/JAK2/STAT3 signaling and enhances MMP activity, thereby activate PI3K/AKT pathway and HIF-1α expression. HIF-1α further induce SPP1 expression in tumor-associated macrophages, promoting M2-like polarization and strengthening macrophage-driven EMT in cancer cells. EMT, epithelial–mesenchymal transition; mTOR, mechanistic target of rapamycin; ERK1/2, extracellular signal-regulated kinase 1/2; GSK-3β, glycogen synthase kinase-3 beta; CD44, cluster of differentiation 44; PTGER4, prostaglandin E receptor 4; TGF-β, transforming growth factor beta; HIF-1α, hypoxia-inducible factor 1-alpha.

The clinical significance of SPP1-mediated EMT is underscored by the strong correlation between SPP1 expression and poor prognosis in various cancers. Meta-analyses have demonstrated that high SPP1 expression is significantly associated with lymph node metastasis, distant metastasis, and reduced overall survival in patients with non-small cell lung cancer, colorectal cancer, and other solid tumors (Song et al., 2024). The combination of SPP1 with other EMT markers, such as vimentin, provides even greater prognostic power, as demonstrated in HCC where the co-expression of SPP1 and vimentin predicts worse overall survival and time to recurrence (Dong et al., 2016) (Figure 2). These findings position SPP1 as a critical driver of EMT and invasion, making it an attractive target for therapeutic intervention aimed at blocking disease progression.

### 3.3 Metastasis

The interaction between SPP1^+^ macrophages and cancer cells is bidirectional and reinforces metastatic progression. Tumor-derived factors play a crucial role in reprogramming resident or infiltrating macrophages towards a pro-metastatic phenotype, often associated with augmented SPP1 expression.

In specific cancer types such as colorectal cancer and oral squamous cell carcinoma, SPP1^+^ macrophages remodel the metastatic lymph node microenvironment by activating fibroblasts through SPP1/CD44 and CD155/CD226 interactions, thereby facilitating metastasis (Zhou et al., 2024; Dong et al., 2024). The SPP1-steroid 5-alpha reductase 3 (SRD5A3) signaling axis has been identified as a crucial driver of lymph node metastasis, through SOX4 transcription factor activation, leading to enhanced mucin-type O-glycan biosynthesis and metastatic capacity (Xu T. et al., 2025). Beyond their role in stromal remodeling, SPP1+ macrophages also exhibit profound metabolic plasticity that is contingent on the specific metastatic niche. For instance, a glycolytic subtype, identified by ATP synthase F1 subunit epsilon (ATP5F1E) expression, was found to be enriched in primary tumors and lymph nodes, whereas an oxidative phosphorylation-driven subtype, associated with Mitochondrially Encoded Cytochrome C Oxidase I (MT-CO1) expression, was predominantly observed in liver metastases (Lin et al., 2026). The SPP1^+^ macrophage subset is enriched in liver metastases compared to primary tumors, and these cells exhibit distinct metabolic and functional properties. This metabolic heterogeneity suggests that SPP1^+^ macrophages adapt to the unique microenvironment of different metastatic sites.

### 3.4 Immune suppression

SPP1^+^ macrophages are potent mediators of immunosuppression within the TME, with the predominant interactive with T cells.

In colorectal cancer liver metastasis, SPP1^+^ macrophages cause exhaustion of tumor-specific CD8^+^ T cells, with metastatic liver tumors containing higher numbers of dysfunctional tumor-specific T cells compared to other organ sites (Trehan et al., 2025). In ovarian cancer, SPP1^+^ TAMs promote T cell exhaustion via the SPP1-CD44 interaction, which emerged as the principal mediator of immune suppression. Blocking either SPP1 or CD44 effectively reversed T cell exhaustion, restored CD8^+^ T cell functionality, and suppressed tumor growth *in vivo* (Hou et al., 2025). This finding highlights the therapeutic potential of targeting the SPP1-CD44 axis to reinvigorate antitumor immunity. On the other side, SPP1^+^ TAMs interact with CD8^+^ exhausted T cells through GDF15-TGFBR2 signaling, which plays a key immunosuppressive role (Du et al., 2024). In addition to direct cell-cell contact, these macrophages secrete soluble factors that impair T cell function. SPP1 itself can directly inhibit T cell activation and proliferation in gastric cancer with liver metastasis (Wang and Denhardt, 2008). In head and neck squamous cell carcinoma, SPP1^+^ macrophages have been shown to increase TNF-α and IL-1β secretion through NF-κB pathway activation, thereby enhancing tumor cell proliferation (Liu et al., 2025).

Furthermore, SPP1^+^ macrophages actively shape the immune landscape by recruiting other immunosuppressive cell types. In bladder cancer, SPP1^+^ macrophages contribute to an immunosuppressive microenvironment by recruiting neutrophils and promoting their pro-tumor functions (Xu W. et al., 2025; Pan et al., 2025). In head and neck squamous cell carcinoma, SPP1^+^ TAMs interact with regulatory T cells to create an immunosuppressive niche via SPP1-CD44 axis that supports tumor progression, further amplifying immunosuppression (Dong et al., 2024; Wu et al., 2024). In pancreatic cancer, the WD repeat domain 5 (WDR5)-H3K4me3 epigenetic axis regulates SPP1 expression in both tumor cells and MDSCs, with SPP1 compensating for PD-L1 function to promote immune escape (Lu et al., 2021). This finding suggests that SPP1^+^ macrophages and MDSCs may cooperate to create redundant immunosuppressive mechanisms that limit the efficacy of immune checkpoint blockade.

Although these pathways represent the major mechanisms by which SPP1 promotes cancer progression by immune regulation, further investigation is warranted to better define these alternative mechanisms and their therapeutic relevance.

## 4 The role of SPP1 in non-cancerous pathologies

The functions of SPP1 extend beyond oncology and encompass important roles in reproductive biology, embryonic development, fibrotic diseases, and neurological disorders. Understanding these non-cancerous functions not only provides insight into the fundamental biology of SPP1 but also has important implications for therapeutic targeting.

### 4.1 Pregnancy-related complications

SPP1 expression in the uterus and placenta is tightly regulated throughout pregnancy, and the functions are essential for successful embryo implantation, decidualization, and the establishment of a functional placenta (Kramer et al., 2021).

During the peri-implantation period, SPP1 is highly expressed in the endometrial luminal epithelium (LE), glandular epithelium, and decidualizing stroma. Uterine spp1 mRNA levels are upregulated on day 4 of pregnancy, corresponding to the window of implantation, and are further increased at implantation sites on days 5 and 8, both in mice and human (Qi et al., 2014; Wang et al., 2018). This temporospatial expression pattern suggests that SPP1 actively participates in preparing the endometrium for embryo attachment and invasion. In pigs and sheep, SPP1 binds to αvβ6 integrin on trophectoderm and αvβ3 integrin on uterine LE, inducing focal adhesion assembly and cell migration through activation of multiple signaling pathways, including PI3K, MAPK, and MTOR, to bridge conceptus attachment to the uterus for implantation (Johnson et al., 2014). In humans, SPP1 promotes trophoblast invasion at least partially through targeting integrin αvβ3, as treatment with an integrin αvβ3 inhibitor significantly attenuates SPP1-enhanced invasive potential of trophoblast cells (Ke et al., 2020).

Beyond implantation, SPP1 expression is upregulated during *in vitro* decidualization through the cAMP and C/EBPβ signaling pathway to support the developing embryo (Wang et al., 2018). In mice, *spp1* mRNA and protein localize to uterine natural killer cells (uNKs) in the decidua, where SPP1 may increase angiogenesis within the decidua to augment hemotrophic support of embryonic development (Kramer et al., 2021). In pigs, SPP1 binds integrins to activate transport of ions across the chorioallantoic placenta, suggesting a role in nutrient and ion exchange between mother and fetus (Wu et al., 2022). SPP1 is also essential for uterine spiral artery remodeling, a process critical for establishing adequate blood flow to the placenta (Pan et al., 2022). During normal pregnancy, extravillous trophoblast cells invade the maternal decidua and remodel the uterine spiral arteries, transforming them into high-capacitance, low-resistance vessels that ensure adequate blood flow to the placenta (Pan et al., 2022). Impaired SPP1 expression or function could lead to inadequate trophoblast invasion and insufficient spiral artery remodeling, resulting in poor placental perfusion and pregnancy complications, including RPL, preeclampsia, and intrauterine growth restriction (Pan et al., 2022; Solt et al., 2025). SPP1 knockout mice exhibit smaller embryos at all gestational ages, although embryo numbers are not significantly affected, suggesting that SPP1 is more critical for optimal fetal growth rather than initial implantation (Weintraub et al., 2004).

**FIGURE 3:**
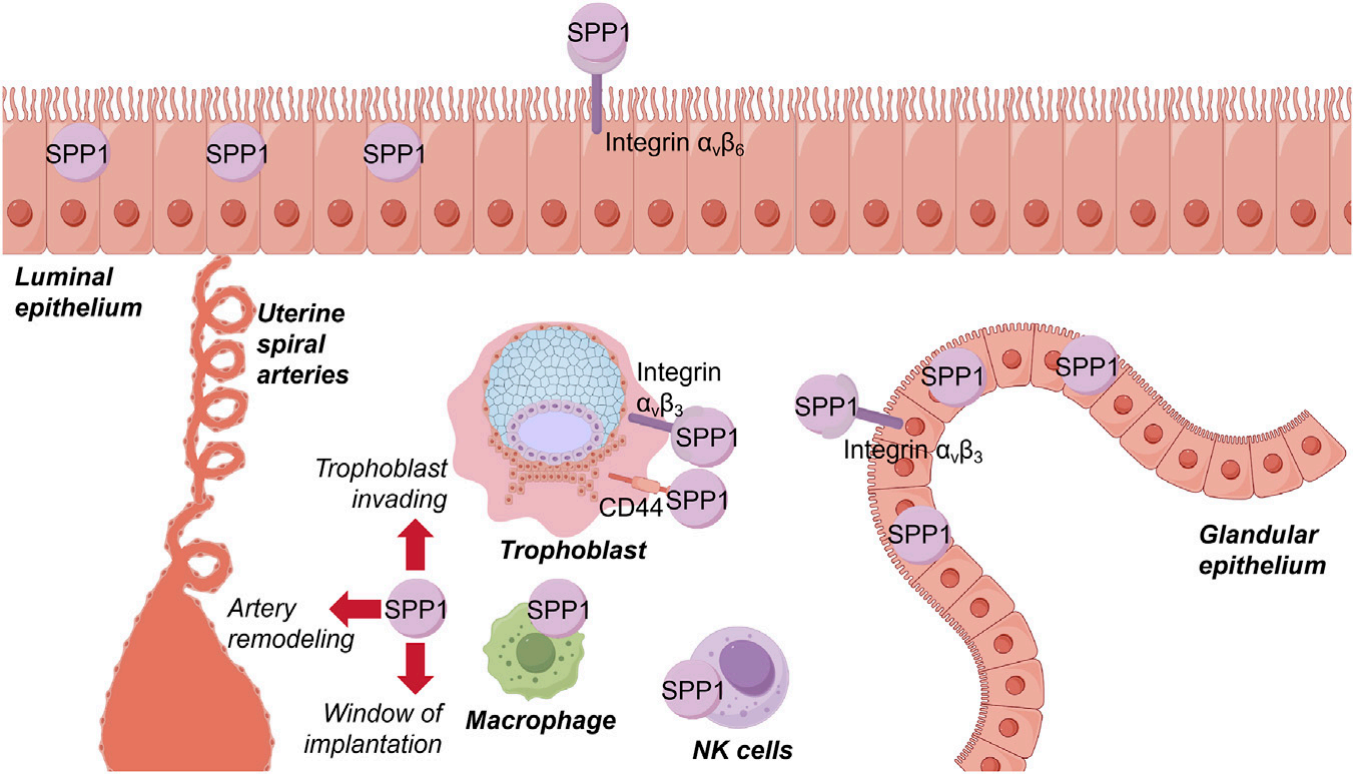
Pleiotropic functions of SPP1 during peri-implantation and pregnancy. SPP1 is spatiotemporally regulated in the luminal and glandular epithelium. SPP1 bridges embryonic attachment via integrin α_v_β_3_/α_v_β_6_ binding to promote trophoblast migration and invasion. Beyond implantation, SPP1 supports pregnancy through: (I) regulating window of implantation; (II) enhancing trophoblast invading; and (III) facilitating angiogenesis and spiral artery remodeling.

Furthermore, SPP1 is a chemoattractant for macrophages and other immune cells and can influence the balance between pro-inflammatory and anti-inflammatory responses at the maternal-fetal interface (Castello et al., 2017; Gu and Muller, 2025). Single-cell analysis of decidual tissues from recurrent miscarriage patients revealed enrichment of inflammatory SPP1^+^ macrophages and alterations in the ECM-CD16^+^ macrophage regulatory axis (Yang Hl et al., 2025; Bao et al., 2023) (Figure 3). These findings suggest that disruption of normal SPP1-mediated macrophage-stromal interactions may contribute to pregnancy loss. The potential of SPP1 as a biomarker for pregnancy loss is an area of active investigation. Further research is needed to fully elucidate the mechanisms by which SPP1 dysregulation contributes to pregnancy loss and to develop safe and effective therapeutic strategies.

### 4.2 Fibrotic diseases

Beyond reproduction, SPP1 exhibits context-dependent pleiotropy in tissue remodeling and fibrotic diseases (Shirakawa and Sano, 2021). In bone homeostasis and neural repair, SPP1 is indispensable for angiogenesis; it enhances vascularization during ectopic bone formation and promotes functional recovery after spinal cord injury via VEGF and AKT pathway activation (Asou et al., 2001; Weng et al., 2024). Conversely, its role in organ fibrosis is dichotomous: while SPP1 supports early wound healing in the heart, its persistent expression drives pathological remodeling (Shirakawa and Sano, 2021; Abdelaziz Mohamed et al., 2019). Similarly, in metabolic-associated fatty liver disease, SPP1 exerts a protective effect by activating the Oncostatin M/STAT3/Arginase2 axis in hepatocytes to augment fatty acid oxidation (Yang J. et al., 2025). However, during chronic injury, SPP1 shifts to a profibrotic phenotype, activating hepatic stellate cells and promoting collagen deposition, thereby driving cirrhosis progression (Yang J. et al., 2025). The dual role of SPP1 in liver disease, with both protective and pathological functions depending on the context, highlights the complexity of targeting this molecule therapeutically. In intrauterine adhesions, SPP1^+^ macrophages play a profibrotic role. Single-cell analysis revealed that macrophage-derived CCL5 and SPP1 promote fibroblast-to-myofibroblast transition, contributing to the development of endometrial fibrosis (Dong et al., 2025). A pH-responsive hyaluronic acid hydrogel delivering an α5β1-selective agonist peptide was shown to shift macrophages from a pro-inflammatory toward a pro-repair program and suppress SPP1 expression, thereby reducing profibrotic signaling and restoring endometrial receptivity (Ji et al., 2026). These findings underscore SPP1 as a critical regulator that balances tissue repair against pathological fibrosis.

### 4.3 Neurological disorders

Beyond the well-characterized roles in pregnancy and fibrosis, emerging evidence implicates SPP1 in the pathogenesis of multiple sclerosis (MS), Alzheimer’s disease (AD), and neurotrauma, highlighting its dual role as both a driver of neuroinflammation and a mediator of neuroprotection.

SPP1 is one of the most consistently elevated proteins in the cerebrospinal fluid and peripheral blood of MS patients. A systematic review and meta-analysis demonstrated significantly higher cerebrospinal fluid and plasma SPP1 levels in MS patients compared with controls, with elevated levels associated with disease activity and progression (Agah et al., 2018). Mechanistically, SPP1 promotes the survival and activation of autoreactive T cells and contributes to the migration of inflammatory cells across the blood-brain barrier. In experimental autoimmune encephalomyelitis, the animal model of MS, SPP1-deficient mice exhibit milder disease severity, reduced T-cell infiltration, and decreased demyelination (Pyka-Fościak et al., 2023). SPP1 has also been investigated as a biomarker of treatment response, natalizumab-treated MS patients who responded to therapy showed decreased plasma SPP1 levels, whereas non-responders maintained elevated levels (González-Jiménez et al., 2025). These findings position SPP1 both as a biomarker for monitoring disease activity and treatment response in MS and as a potential therapeutic target.

In AD, SPP1 exhibits a complex, context-dependent role. SPP1 is upregulated in the hippocampus of AD patients and colocalizes with amyloid-β (Aβ) plaques and neurofibrillary tangles (Sung et al., 2023). Cerebrospinal fluid levels of SPP1 and its thrombin-cleaved fragments have been identified as biomarkers predicting the progression from mild cognitive impairment to AD dementia (Azizan et al., 2025). At the cellular level, single-cell and spatial transcriptomic analyses have identified distinct SPP1-expressing microglial subpopulations with divergent functional consequences. The SPP1^+^CD11c^+^ cells is known to be associated with microglia activation and neuroinflammatory diseases, whereas SPP1^+^CD11c^+^ microglia exhibited a pro-inflammatory phenotype with impaired Aβ uptake and were enriched three-fold in AD brains compared with controls (Lopes et al., 2024). Furthermore, perivascular macrophage-derived SPP1 has been shown to drive pathological microglial synaptic engulfment, contributing to synapse loss, a hallmark of early AD (De et al., 2023). Conversely, systemic monocyte-derived SPP1 has been reported to facilitate Aβ clearance and tissue repair in late-stage AD, suggesting that the cellular source of SPP1 critically determines its functional outcome. Anti-SPP1 monoclonal antibodies have demonstrated the capacity to reduce pro-inflammatory microglia and Aβ plaque burden in AD transgenic mouse models, supporting the therapeutic potential of targeting SPP1 in neurodegeneration (Xu et al., 2026).

The collective evidence underscores SPP1 as a significant mediator of neuroinflammation across a range of neurological disorders. Its dual functionality mirrors the pattern observed in cancer and fibrosis. Future research should aim to evaluate whether SPP1-directed therapies developed for oncology and fibrosis can be safely repurposed for neurological indications.

## 5 Therapeutic strategies and opportunities in precision medicine

**FIGURE 4:**
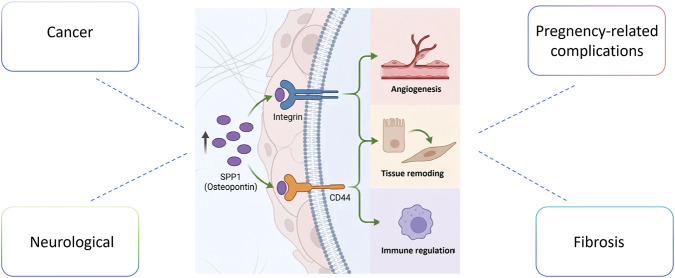
Roles of SPP1 across disease contexts. SPP1 serves as a central molecular hub that orchestrates three biological processes, including angiogenesis, tissue remodeling, and immune regulation, through integrin and CD44 receptor engagement. These shared mechanisms underpin the pathological roles of SPP1 in cancer, pregnancy-related complications, fibrotic diseases, and neurological disorders.

Understanding the multifaceted roles of SPP1 across diverse disease contexts (Figure 4) is beneficial for highlighting its potential utility as both a prognostic biomarker and a promising therapeutic target for precision medicine approaches.

The association of SPP1 expression in blood and other body fluids with poor clinical outcomes across multiple cancer types has generated significant interest in SPP1 as a biomarker and therapeutic target (Dong et al., 2021; Son et al., 2023). The development of SPP1-targeted interventions, including neutralizing antibodies and aptamers, therefore offers new therapeutic opportunities for angiogenesis-dependent diseases (Wang et al., 2026; Droho et al., 2023). The emergence of precision medicine approaches, including the characterization of SPP1 splice variants and the identification of SPP1^+^ immune cell subsets, offers new opportunities for targeted therapeutic interventions (Hatipoglu et al., 2021; Tang et al., 2021; Zeng et al., 2022). Three major splice variants have been identified, including OPN-a (full-length), OPN-b (lacking exon 5), and OPN-c (lacking exon 4) (An et al., 2023; Anborgh et al., 2011). Differential expression of these isoforms influences integrin-binding preferences, susceptibility to proteolytic cleavage, and downstream signaling pathway activation. Furthermore, post-translational modifications (phosphorylation, glycosylation, sulfation) and proteolytic processing by thrombin and matrix metalloproteinases generate functionally distinct SPP1 fragments with divergent biological activities. The identification of SPP1 splice variants has opened new avenues for precision medicine approaches (Anborgh et al., 2011). Another critical unresolved question in SPP1 biology is whether its actions are primarily exerted in a paracrine or autocrine manner, and how this distinction bears on therapeutic strategy design. Therapeutic strategies targeting autocrine SPP1 would ideally focus on inhibiting SPP1 synthesis or secretion by the malignant cells themselves, or by blocking SPP1 receptors on the tumor cell surface. Conversely, in a paracrine fashion, direct targeting of the cells or SPP1 receptors might be most effective. Elucidating the dominant mode of action in specific disease contexts through detection and cell-type-specific perturbation experiments is paramount for rational therapeutic development. Combination of SPP1 targeting with other therapeutic modalities, including chemotherapy, radiotherapy, and immunotherapy, holds promise for enhancing treatment efficacy and overcoming resistance (Pang et al., 2021).

To systematically evaluate the current landscape, we have compiled a comprehensive overview of these approaches, detailing their mechanisms and associated hurdles (Table 1). Despite the promise of SPP1-targeted therapies, several challenges remain. Systemic inhibition of SPP1 could potentially lead to adverse effects, including impaired bone healing, increased susceptibility to infections, or delayed wound healing. Therefore, careful consideration must be given to the dosing, timing, and duration of SPP1-targeted therapies to minimize off-target effects.

**TABLE 1:** Therapeutic strategies targeting the SPP1 signaling.

SPP1 targeted strategy	Agent(s)	Mechanism	Current evidence	Limitations
Neutralizing Monoclonal Antibodies	Anti-SPP1 mAb (AOM1)	Block SPP1 binding to integrins (αvβ3, αvβ5, α9β1) and CD44; inhibit downstream PI3K/AKT, ERK, and NF-κB signaling	AOM1 suppressed lung metastasis (Shojaei et al., 2012)	High plasma concentrations require high antibody doses; standard antibodies achieve only 20% free SPP1 reduction
Aptamers/RNA Aptamers	RNA aptamer OPN-R3; DNA aptamers	High-affinity binding to SPP1, sterically blocking receptor interaction sites; nuclease-resistant modifications enhance stability	OPN-R3 aptamer attenuated liver progenitor cell response and fibrosis in 3 mouse liver injury models; suppressed tumor growth and metastasis in breast cancer xenografts (Li et al., 2026)	Rapid renal clearance; limited *in vivo* stability; delivery and tissue penetration challenges; no clinical trials reported to date
Integrin-Blocking Peptides	7aaRGD; RGD-based cyclic peptides	Competitive inhibition of SPP1 binding to RGD-recognizing integrins; disrupt outside-in integrin signaling	7aaRGD reversed immunosuppression and improved anti-PD-1 immunotherapy outcomes in experimental gliomas (Ellert-Miklaszewska et al., 2025)	RGD peptides may cross-inhibit other RGD-containing ECM proteins; limited pharmacokinetic half-life
siRNA/shRNA/RNA Interference	SPP1-targeting siRNA; SPP1 shRNA lentiviral vectors	Post-transcriptional silencing of SPP1 gene expression; reduces both intracellular and secreted SPP1 protein	SPP1 silencing attenuated bleomycin-induced pulmonary fibrosis by regulating EMT (Hatipoglu et al., 2021); SPP1 knockdown reduced MMP-2/uPA expression and vasculogenic mimicry in HCC (Liu et al., 2011); inhibited enzalutamide resistance in CRPC via PI3K/AKT and ERK1/2 suppression (Pang et al., 2021)	Delivery barriers (tumor penetration, cellular uptake); off-target effects; transient suppression; immunogenicity of delivery vectors
Small-Molecule Inhibitors	CD44 antagonists; HIF-1α inhibitors; PI3K/AKT pathway inhibitors	Indirect targeting of SPP1 downstream signaling; block SPP1-CD44 interaction; inhibit SPP1 transcriptional regulators; prevent SPP1-mediated pathway activation	CD44 blockade reversed T cell exhaustion in ovarian cancer models (Hou et al., 2025); HIF-1α inhibition reduced SPP1 expression and EMT in colorectal cancer (Qu et al., 2025)	Lack of direct SPP1 specificity; complex compensatory signaling networks; systemic toxicity from CD44 blockade (CD44 is widely expressed)
Combination Strategies	Anti-SPP1 immune checkpoint inhibitors (anti-PD-1/PD-L1); Anti-SPP1 chemotherapy (cisplatin, carboplatin)	Simultaneously target SPP1-driven immunosuppression and PD-1/PD-L1 axis; sensitize chemoresistant tumors by disrupting SPP1-mediated survival signaling	Anti-SPP1 enhanced anti-PD-1 immunotherapy in glioma models (Ellert-Miklaszewska et al., 2025); SPP1 knockdown sensitized lung cancer cells to cisplatin (Tang et al., 2021); AOM1 ^+^ carboplatin showed synergistic inhibition of lung metastasis in GEMM. SPP1 may compensate for PD-L1 function in pancreatic cancer immune escape (Lu et al., 2021)	Optimal sequencing and dosing regimens undefined; potential for compounded toxicity; patient stratification biomarkers needed; tumor-type-specific responses

## 6 Conclusion and future perspectives

SPP1 has emerged as a pivotal molecular hub linking a broad spectrum of biological processes across both physiological and pathological settings. Through its capacity to engage multiple integrin receptors and CD44 variants, SPP1 orchestrates diverse downstream signaling networks to associate with cell adhesion, migration, survival, immune modulation, and tissue remodeling. The included studies in this review exhibited substantial variability in design, SPP1 detection methodologies, and sample types. These methodological disparities probably limit the direct comparability of findings. Future studies should aim to integrate mechanistic investigation with translational research in order to identify clinically actionable vulnerabilities. Ultimately, such efforts may enable the safe and effective exploitation of SPP1 as both a biomarker and a therapeutic target, thereby improving clinical outcomes in cancer and potentially other SPP1-associated diseases.
